# Circulating Tumor Cells in the Adenocarcinoma of the Esophagus

**DOI:** 10.3390/ijms17081266

**Published:** 2016-08-04

**Authors:** Giulia Gallerani, Francesco Fabbri

**Affiliations:** Biosciences Laboratory, Istituto Scientifico Romagnolo per lo Studio e la Cura dei Tumori (IRST) IRCCS, Via P. Maroncelli 40, Meldola 47014, FC, Italy; francesco.fabbri@irst.emr.it

**Keywords:** circulating tumor cells, esophagus adenocarcinoma, liquid biopsy

## Abstract

Circulating tumor cells (CTCs) are elements of indisputable significance as they seem to be responsible for the onset of metastasis. Despite this, research into CTCs and their clinical application have been hindered by their rarity and heterogeneity at the molecular and cellular level, and also by a lack of technical standardization. Esophageal adenocarcinoma (EAC) is a highly aggressive cancer that is often diagnosed at an advanced stage. Its incidence has increased so much in recent years that new diagnostic, prognostic and predictive biomarkers are urgently needed. Preliminary findings suggest that CTCs could represent an effective, non-invasive, real-time assessable biomarker in all stages of EAC. This review provides an overview of EAC and CTC characteristics and reports the main research results obtained on CTCs in this setting. The need to carry out further basic and translational research in this area to confirm the clinical usefulness of CTCs and to provide oncologists with a tool to improve therapeutic strategies for EAC patients was herein highlighted.

## 1. Introduction

Esophageal carcinoma (EC) is one of the most common malignant tumors in the world and the sixth most common cause of death from cancer, with estimated 400,000 deaths in 2012 (4.9% of the total) and an overall 5-year survival rate ranging from 15% to 25% [1,2,3]. From the second half of the 1990s to the early 2000s the age-adjusted incidence of EC in Europe increased by 39.6% for men and 37.5% for women [4], indicating an alarming trend. EC is an extremely aggressive tumor characterized by very poor survival rates and by an epidemiologic pattern distinct from other cancers [5,6]. It is often diagnosed at an advanced stage (40% of patients), when the 5-year survival rate is lower than 3% [1,7,8]. EC typically occurs in one of two forms: squamous cell carcinoma (ESCC), arising from the stratified squamous epithelial lining of the organ, or adenocarcinomas (EAC) in which columnar glandular cells replace the squamous epithelium [9]. Despite intense research efforts, the pathogenesis of this disease is still widely debated. It is generally accepted that EAC is a direct consequence of the condition known as Barrett’s esophagus (BE), a condition in which the stratified epithelium is substituted by metaplastic columnar epithelium [10]. However, only a small fraction of individuals with BE go on to develop esophageal adenocarcinoma (about 0.22%) [11]. Conversely, some patients are diagnosed with EAC despite no prior finding of BE [12]. At present, repeated endoscopic biopsies with histological evaluation of dysplasia is the only means of evaluating the risk of progression to cancer. However, such an approach is hampered by all the challenges of these clinical determinations, e.g., inter-observer differences and lack of reproducible diagnostic classification [13], highly heterogeneous dysplasia in terms of progression to adenocarcinoma [14], or high number of biopsies required to reduce the risk of sampling error caused by primary tumor heterogeneity [15,16]. Moreover, once EAC has been diagnosed, there are few reliable methods to stratify patients and identify the most suitable therapeutic approach [17]. Thus, new reliable and reproducible diagnostic, prognostic and predictive markers must urgently be sought to improve the overall management of EAC patients.

Circulating tumor cells (CTCs) are exceedingly rare, genetically and phenotypically heterogeneous cells found in the peripheral blood of cancer patients. Their presence is correlated with poor prognosis and progression-free survival, and they are considered indicators of treatment efficacy in different tumors [18,19,20,21]. CTCs can be considered as precursors of metastatic dissemination, one of the key element of the metastatic process, and are one of the main elements of liquid biopsy. However, their clinical use for tumor staging, disease monitoring and choice of treatment has yet to become a reality. Despite this, CTCs have the potential to serve as a biopsy for the “leukemic phase” of solid tumors [22]. Liquid biopsies enable patients with early or advanced disease to be stratified into prognostic groups. They could also potentially be used as a surrogate endpoint of survival for studies on therapeutic efficacy and for the molecular sub-classification of advanced cancer patients [23,24]. Thus, they represent a real opportunity to bring the science of personalized medicine to realization in clinical practice.

This review focuses on the biology and clinical characteristics of EAC and on preliminary findings and potentialities of the use of CTCs as prognostic and predictive marker in this tumor. It also highlights the ability of CTCs to provide a real-time snapshot of tumor features (heterogeneity, aggressiveness, invasion capacity, etc.) that could be capable of offering clinically relevant information. An English-language literature search using PubMed/MEDLINE (up to the end of June 2016) was performed using the key words CTCs, CTC detection methods, EAC, survival, prognosis, progression, EAC-pathogenesis and EAC-staging system. All pertinent articles containing human clinical trial data and relevant information were evaluated and included if appropriate.

## 2. Esophageal Adenocarcinoma (EAC) Pathogenesis and Features

One of the first steps in the development of EAC is the transition from normal esophageal epithelium to columnar and secretory epithelium, a process often associated with chronic inflammatory events triggered by gastro-esophageal reflux. The genesis of metaplasia is thought to be a response to chronic tissue inflammation [25] and is known as Barrett’s esophagus (BE) [26,27,28]. Although a number of papers has been published on this topic, there is still relatively little information available about tissue homeostasis in the epithelium of the esophagus. Furthermore, the role of candidate stem cells in the growth and regeneration of this tissue has not yet been fully defined in vivo [29]. However, research has shown that esophageal epithelium is maintained by a population of cells capable of both maintaining and repairing tissue and which divide to produce proliferating and differentiating daughter cells with equal likelihood, without the need for a slow-cycling stem cell pool [30]. Although in contrast to previous conventional theories regarding stem cells [31], this information is probably a key to identifying the mechanisms of pathogenesis of esophageal cancer and may also help to clarify the genetic heterogeneity of EAC. Recent findings on genomic abnormalities in EAC, occurring the early stages of disease [16,32], include conventional single nucleotide variants in 26 genes, recurrent deletions and focal amplifications, and mutations in chromatin-remodeling genes [33,34,35,36]. Within this context the linear model of carcinogenesis may not optimally define BE to EAC progression as it does not explain the genetic heterogeneity of EAC. Findings similar to those observed in breast and colorectal cancer [37] have demonstrated that all cancers are probably a spectrum of diseases and as such, EAC is no exception [17]. In addition, BE genetic heterogeneity may explain EAC clonal diversity and may predict transformation to adenocarcinoma [32]. Thus, the current challenge is to identify and validate new diagnostic, prognostic and predictive biomarkers that can unveil EAC genetic and cellular heterogeneity and, in doing so, help clinicians to select the best treatment option.

CTCs are “multifaced” cells that actively or passively leave the primary tumor, follow potential dissemination pathways, and are able to reach distant localizations and adapt to different microenvironments [38,39]. They are a remarkably rare and heterogeneous cell population at the genetic and phenotypic level and potentially composed of a combination of subpopulations with dissimilar features [40]. An interesting hypothesis to explain the starting point of CTC spread and the mechanisms involved are those of the epithelial to mesenchymal transition (EMT) process [41,42]. EMT is considered a normal phenomenon implicated in embryogenesis and wound healing processes that may be activated during cancer progression and metastasis [43,44,45]. The process induces a significant change in cell phenotype associated with aggressive biological behavior in cancer cells, loss of cell junctions [46] and of apical-basal polarity [47], and enhanced CTC motility which facilitates intravasation into the bloodstream. EMT and its reverse process, mesenchymal to epithelial transition (MET), could enable CTCs to switch backwards and forwards between phenotypes, causing resistance to anoikis, to the physical stress induced by blood circulation, and also to chemo- and radio-therapy [43,48]. As EAC is a paradigm for inflammation-associated cancer [49], and inflammation processes have been reported to contribute to tumor progression, metastasis, EMT [50] and CTC spreading, it is tempting to hypothesize that an almost direct connection exists between all of these phenomena. Taken together, these hypotheses indicate that CTCs could be an extremely effective biomarker of EAC in terms of its genetic heterogeneity, clonal evolution and sensitivity or resistance to treatment. In the near future, once technical and methodological aspects of the CTC research area are sufficiently sensitive, specific and representative of systemic disease, these circulating cells could become a significant biomarker in EAC.

## 3. EAC Clinical Aspects

Esophageal cancer staging has been defined by the American Joint Committee on Cancer (AJCC) Staging System which established a tumor-node-metastasis (TNM) classification with sub-classifications based on the depth of invasion of the primary tumor (T), lymph node involvement (N), and extent of metastatic disease (M) [51]. Current management of EAC is mainly based on complete preoperative assessment because accurate pre-treatment staging and subsequent stage-appropriate treatment is crucial to optimize EAC outcome. Diagnosis is made mainly by endoscopy and often includes multiple biopsies of the upper digestive tract. Once EAC has been histologically confirmed, clinical stage is determined by further instrumental tests. A computerized tomography (CT) scan provides valuable information about the longitudinal extension of the tumor and is useful in identifying the presence of distant metastases such as those of the lung and liver but somewhat limited in defining nodal involvement [52]. Positron-emission tomography (PET) scans represent an important aid to staging in that they detect previously unseen metastatic disease in up to 15%–20% of cases [53,54]. However, PET is generally considered a more suitable instrument for post-treatment assessment, especially that of neo-adjuvant therapy [55]. Despite these strategies, small secondary lesions may nevertheless be missed and patients may also have undetected pleural or peritoneal disease [56]. A more accurate staging system is thus urgently required to improve treatment strategies from the early stages of disease onwards. Novel tools for early tumor detection, adequate prognostic staging, and accurate treatment monitoring and selection, in particular in the neo-adjuvant setting, are also needed. A new staging category, M0(i+), was recently proposed by the AJCC [57]. M0(i+) is defined as “no clinical or radiographic evidence of distant metastases, but deposits of molecularly or microscopically detected tumor cells detected in circulating blood, bone marrow, or other non-regional nodal tissues, that are no larger than 0.2 mm in a patient without symptoms or signs of metastases”. This new M category could significantly improve cancer staging and consequently the therapeutic management of the disease. Using M0(i+) staging in EAC could also help to overcome the limitations of CT and PET in detecting minimal occult disease, often defined by the presence of CTCs in the blood.

## 4. Circulating Tumor Cells (CTCs) and EAC

Recent advances in techniques to identify CTCs in the blood of patients with different types of cancer have generated interesting results. However, there is still a lack of methodological uniformity and a relatively high variability in detection rates, probably due to the markers used for CTC identification. The most widely used definition of a CTC, i.e., an EpCAM^+^/CK^+^/CD45^−^ cell with a round or oval intracellular nucleus [23], refers mainly to epithelial markers, especially on EpCAM. However, this description falls short of including many potential CTC markers, subpopulations and clusters [40]. Further approaches that permit the identification of EpCAM-negative cells are thus urgently needed [58,59,60,61]. Non-EpCAM-based and EMT-related methods will be of unquestionable importance to decrease the risk of EpCAM-based analysis. In order to improve results, a combination of markers and enrichment strategies to identify EpCAM-negative cells is also needed. Immunocytological, molecular, densitometric and/or size-dependent CTC enrichment methods could lead to significantly different results from those obtained by EpCAM-based technologies [58,59,60,61,62,63]. Approaches that combine physical and cellular properties of CTCs have produced interesting results [64,65,66], as previously reported by our group [67,68]. Our approach, albeit improvable, was based on densitometric enrichment followed by an immunocytological detection step and allowed us to detect three CTC classes: EpCAM^+^/CKs^+^, EpCAM^−^/CKs^+^ and EpCAM^+^/CKs^−^ cells. Although it was not possible to clearly distinguish between these three classes, the method enabled us to specifically detect a more wide-ranging “epithelial” phenotype. It can also detect CTCs that are positive for a “single marker”, such as EpCAM^−^/CKs^+^ or EpCAM^+^/CKs^−^ cells, permitting the identification of EpCAM^−^ cells and reducing the risk of inadequate EpCAM-based enrichment, which may lead to an underestimation of the significance of CTCs [58]. In a gastro-esophageal cancer setting, Kubisch et al. [69] reported an immuno-magnetic enrichment that included mucin-1 in addition to EpCAM, suggesting that a single marker is not enough to identify all CTCs.

Despite these limits, preliminary studies on CTC status before and after surgery in patients with esophageal squamous cancer showed that the presence of CTCs was an independent predictor of disease recurrence [70]. Using reverse transcriptase-polymerase chain reaction assay, CTC detection rates ranging from 2% to 32.9% were found in patients [70,71]. Since these pioneering works were published, very few other investigations have been conducted in this setting. During this time, CTCs specifically classified as EpCAM^+^/CK^+^/CD45^−^ cells and identified by the CELLSEARCH^®^ System (Jannsen Diagnostics, Raritan, NJ, USA) have been definitively shown to be independent predictors of progression-free survival (PFS) and overall survival (OS) in patients with other metastatic cancers, e.g., breast cancer [72]. To date, the CELLSEARCH^®^ System is the only standardized FDA-approved device and whose clinical validity has been confirmed. In 2008, Hiraiwa et al. [73] observed that about 21% (5/23) of patients with metastatic EC were CTC-positive and had a significantly shorter overall survival than CTC-negative cases. However, the number of patients was too small for the study to reach statistical significance. In 2013, a small pilot study on a cohort of 18 patients with advanced esophago-gastric cancer carried out using the CELLSEARCH^®^ System reported that 44% of patients showed >2 CTCs/7.5 mL of blood before first-line chemotherapy [74]. Only 11 of the 18 patients had cancer of the esophagus itself (9 esophagogastric junction, 2 esophagus). Among these, only 4 with esophagogastric disease had >2 CTCs/7.5 mL of blood, whereas both cases of esophageal cancer showed ≤2 CTCs. Although this study confirmed the feasibility of CTC research in this clinical setting, it failed to draw any definitive conclusions about the relationship between CTCs and esophageal cancer due to the low number of cases studied. A larger, well-defined study to assess CTCs as a staging tool for non-metastatic esophageal cancer in prognostic subgroups was carried out by Reeh et al. [75]. The study enrolled 100 patients, including 29 with squamous cell carcinoma (SCC), 68 with adenocarcinoma, 2 with anaplastic carcinoma and 1 with a mixed-type cancer. Using a cutoff of one or more CTCs, the authors found that CTC-positive patients with non-metastatic disease had a significantly shorter overall and relapse-free survival than those without CTCs. This research demonstrated the clinical significance of CTCs as a preoperative staging factor in EAC, independently of other risk indicators such as histological subtype, tumor stage, lymph node (LN) invasion, and tumor grade. Interestingly, only 3 (10.3%) of the 29 patients with ESCC showed ≥1 CTC, whereas 14 (20.6%) of the 68 patients with EAC showed ≥1 CTC. This result could be due to dissimilar characteristics between esophageal cancer subtypes (SSC vs. EAC), e.g., varied EpCAM expression resulting in diverse CTC detection rates, indicating a potentially clinically relevant difference between the two histotypes [75]. Consistent with this hypothesis, an interesting study by Driemel et al. [76] reported that EpCAM expression in disseminated tumor cells (DTC) in early esophageal cancer may vary. In their study, analysis of EpCAM status in DTCs derived from lymph nodes and bone marrow showed that CK18-positive DTCs often lack EpCAM expression. CK18^+^ DTCs were detected in 38.9% of esophageal cancer patients, but co-expression of EpCAM was seen in only 37.1% of DTC-positive cases, whereas 62.9% of patients showed CK18^+^/EpCAM low/negative DTCs. A comparison of EpCAM expression in 14 pairs of primary tumors and their associated DTCs revealed an absence of EpCAM in DTCs in 64% of patients with EpCAM-overexpressing primary tumors. It was concluded that this discrepancy was not due to an intrinsic characteristic of the primary tumor, but most probably to EpCAM knockdown and/or EMT induction. Hence, it can be hypothesized that the metastatic progression of esophageal cancer may be supported by both EpCAM-positive and EpCAM-low/negative cancer cells in a context-dependent manner, and that this different phenotype may also be histotype-specific. Taking together, these findings suggest that CTC studies involving the CELLSEARCH^®^ System may be limited due to the EpCAM down-regulation observed in DTCs. In agreement with literature data, EpCAM should not to be used as the only CTC identification marker as it may underestimate the actual amount of circulating and/or disseminated tumor cells. Although less specifically targeted at EAC, but in line with the results obtained by Reeh and et al., the paper by Kubisch et al. [69] suggests that the presence of CTCs is a predictor of outcome in patients with gastro-esophageal neoplasia, supporting the role of this biomarker as a marker of survival and progression in this setting. CTCs and DTCs could help to unravel the genetic heterogeneity of the tumor during its evolution. Monitoring genetic heterogeneity could provide significant information about cancer progression, potential new therapeutic targets, and tumor sensitivity or resistance to therapy. However, single tumor cell isolation and sequencing are still substantially hampered by methodological challenges such as cell isolation and manipulation, whole genome amplification, and genome-wide analysis [77]. Up to now, in addition to primary tumor tissue analysis, genetic heterogeneity in EAC has mainly been dissected in DTCs. Stoecklein et al. [78] observed that DTCs from lymph nodes and bone marrow differed from those of primary tumors in their genetic aberrations. The only commonly conserved region in cells disseminated lymphatically and hematogenously was the chromosomal region comprising *HER2*. The gain in just one DTC was enough to confer a poor prognosis. In agreement with a functional study on cell lines isolated from DTCs and primary tumors, data reported in the paper by Stoecklein et al. were suggestive of a new drug target during disease evolution. This has led to the possibility of extrapolating new common patterns of targetable alterations through the study of genetic heterogeneity. Despite the importance of such results, technical limitations still exist, hindering their implementation into clinical practice. In particular aCGH analysis which has a limited resolution hence it and may therefore have missed small genetic alterations and the real extension of the overall genetic heterogeneity. Next-generation sequencing (NGS) approaches developed more recently could potentially help to identify new and smaller genetic changes also [79,80] even in CTCs as well [81,82,83], paving the way towards the use of DTCs and CTCs as more effective tools to select treatments and to monitor disease progression and relapse.

## 5. Conclusions

The usefulness of CTCs is still very much open to debate and their full implementation into clinical practice is unlikely in the near future. The lack of technical standardization for their identification, as well as limited data on their cellular and molecular characteristics, represent significant obstacles to the recognition of the clinical utility of this marker. Despite this, CTCs still hold great potential [84]. The detection of minimal occult disease is still an unmet clinical need in EAC and liquid biopsy could prove to be the solution to this problem. As already hypothesized by some authors, liquid biopsy could help us to better understand and manage EAC in terms of disease monitoring, choice of therapy and identification of drug resistance [85,86]. As already suggested [87], it is appealing to imagine that in the EAC setting, CTCs could be utilized for diagnosis, drug resistance identification, monitoring of occult disease, early relapse, and therapeutic efficacy, and selection of targeted drugs. However, their evaluation still presents several problems, especially with regard to their actual value and how they should be investigated, i.e., usefulness of evaluation of single CTCs, optimal number of cells to be tested, cell subpopulations worthy of a more in-depth analysis. Further studies are warranted to address these issues in an attempt to bring CTCs from the bench to the bedside in EAC.
